# Predictors and oncological outcomes of achieving Pentafecta in radical cystectomy: a meta-analysis

**DOI:** 10.3389/fonc.2025.1682830

**Published:** 2026-01-08

**Authors:** Zhiqiang Zeng, Wubin Chen, Wangbing Chen, Lunhong Zou, Tao Li, Tao Zhou, Huan Zhao, Xionglin Hu, Peng Ji, Yang He, Yubo Zhou

**Affiliations:** 1 Santai People‘s Hospital (Santai Hospital Affiliated to North Sichuan Medical College), Mianyang, Sichuan, China; 2 North Sichuan Medical College (University), Nanchong, Sichuan, China

**Keywords:** meta-analysis, oncological outcomes, Pentafecta, perioperative outcome, radical cystectomy

## Abstract

**Objective:**

This study aims to evaluate the predictive value of RC-Pentafecta on overall survival (OS) and cancer-specific survival (CSS) after radical cystectomy (RC) through meta-analysis and to explore the perioperative predictors of RC-Pentafecta.

**Method:**

This systematic review and meta-analysis were conducted in accordance with the PRISMA statement. We systematically searched PubMed, Embase, Cochrane Library, and Web of Science databases and included 9 retrospective studies from 2020 to 2025. The fixed effect model and random effect model were used for combined analysis.

**Results:**

The results of the study showed that patients who achieved RC-Pentafecta had significantly better OS and CSS than those who did not. In addition, Age, length of hospital stay (LOS), American Association of Medical Sciences Anesthesiology (ASA) score, diabetes mellitus, hypertension, Type of Urinary diversion (UD), pathological T-stage (pT), and pathological N-stage (pN) showed significant differences among the groups that achieved RC-Pentafecta.

**Conclusions:**

RC-Pentafecta is a valuable criterion that can effectively predict OS and CSS in patients after RC. Age, perioperative health status, and pathological stage are important predictors of RC-Pentafecta.

## 1 Introduction

Bladder cancer (BCa) is the 11th most common cancer worldwide, and its incidence and mortality have been increasing in recent years (1, 2). Radical cystectomy (RC) is the standard treatment for high-risk non-muscle invasive and muscle invasive BCa. However, due to the complexity of the operation and the many postoperative complications, it is of great clinical significance to evaluate the success of the operation and the long-term oncological outcome of the patient (3–8). Traditional evaluation criteria, such as trifecta, are commonly used to predict short-term perioperative outcomes, including 1. negative soft tissue surgical margins (STSMs), 2. ≥16 lymph node (LN) yield, 3. absence of major (grade III-IV) complications at 90 days. However, these scoring systems do not fully consider the long-term oncological outcome and functional recovery after surgery, especially for the prediction of patient survival and recurrence risk (9, 10). Therefore, there is a need for more integrated and comprehensive criteria for surgical success that accurately reflect oncological and functional outcomes after surgery. Researchers have proposed Pentafecta criteria. Pentafecta is a comprehensive evaluation system that integrates short-term surgical safety, long-term tumor control, and postoperative functional recovery on the basis of the traditional Trifecta of short-term surgical quality only. All included studies adopted a unified five compliance criteria, as detailed below: (1) negative soft tissue surgical margins (STSMs), (2) ≥16 lymph node (LN) yield, (3) absence of major (grade III-IV) complications at 90 days, (4) absence of UD-related long-term sequelae, 5. the absence of clinical recurrence at ≤12 months. It should be noted that all five indicators of Pentafecta must be met simultaneously to be considered up to standard. Failure to meet any one of the indicators will be regarded as non-compliance (11, 12). Previous studies have shown that RC-Pentafecta is closely related to better oncological outcomes, such as cancer-specific survival (CSS) and overall survival (OS) (12, 13). However, Baron et al. (14) found that RC-Pentafecta did not significantly improve OS in European patients.

As no relevant meta-analysis was found. Therefore, it is necessary to conduct a meta-analysis to comprehensively evaluate the ability of RC-Pentafecta to predict oncological outcomes in patients with RC and the predictors of achieving RC-Pentafecta perioperation. To provide evidence-based recommendations for its use in clinical practice.

## 2 Methods

### 2.1 Literature search

We conducted a comprehensive review and a meta-analysis of key outcomes in line with the PRISMA criteria (15, 16) and AMSTAR guidelines (assessing the methodological quality of systematic reviews) (17). This review is registered with PROSPERO.

Two investigators independently executed the literature search and screening; in cases of disagreement, a third reviewer was consulted to resolve the dispute. Four databases were searched: Embase, PubMed, Cochrane Library, and Web of Science. The search period was from the creation of each database to May 2025. Search terms included: Pubmed: “Pentafecta” and (“cystectomy” or bladder); Embase: ‘Pentafecta’/exp and (‘Cystectomy’/exp OR ‘Cystectomies’ OR ‘Partial Cystectomy’ OR ‘Radical Cystectomies’ OR ‘Cystectomy, Radical’ OR ‘‘); Cochrane Library: ((Cystectomy):ti,ab,kw OR (Cystectomies):ti,ab,kw OR (Partial Cystectomy):ti,ab,kw OR (Radical Cystectomies):ti,ab,kw OR (Cystectomy, Radical):ti,ab,kw) AND Pentafecta; Web of Science: Pentafecta(Topic) AND [cystectomy(Topic) OR bladder(Topic)]. Manually searching related research references to expand the search scope. We also searched grey literature, such as unpublished research reports, conference abstracts, and other similar materials, and trial registration platforms. No language restrictions were applied to the search. Two authors independently reviewed article titles and abstracts for eligibility, and divergences were settled by consensus. Hand-searching reference lists of relevant studies broadened the scope of the search. PROSPERO registration number: CRD42024578765.

### 2.2 Eligibility criteria

Reports were included in our systematic review if they met the inclusion criteria: (1) Studies involving RC; (2) Patients were grouped according to whether they achieved RC-Pentafecta or not; (3) Contained at least one oncology outcome, such as OS, RFS, CSS. Or include perioperative predictors of achieving RC-Pentafecta. We excluded studies based on the following criteria: (1) Those from which relevant data could not be retrieved; (2) Publications that were editorials, conference proceedings, or expert commentaries; (3) Research with duplicated participant data presenting identical outcomes; (4) Investigations involving non-human participants; (5) Studies that did not make a comparison by RC-Pentafecta.

### 2.3 Data extraction

Two independent reviewers independently selected articles for inclusion and extracted the data using a pre-established data collection form. Extracted data included author, year of publication, sample size, age, document type, Body Mass Index (BMI), tumor size, pathological T/N staging, operative time, length of hospital stay(LOS), OS, CSS, RFS, type of urinary diversion (UD), smoking, American Association of Anesthesiology (ASA) score, neoadjuvant chemotherapy (NAC), diabetes, hypertension.

### 2.4 Study quality assessment

Retrospective studies were assessed using the Newcastle-Ottawa scale (NOS) (18). The NOS scores range from 0-9, with more than 6 being high quality.

### 2.5 Risk of bias assessment

Two researchers independently evaluated the risk of bias within the selected studies using the ROBINS-I tool, designed for non-randomized studies. This tool examines seven key areas of potential bias: confounding, selection, classification of intervention, deviations from the protocol, missing data, measurement of outcomes, and selection of reported results. Each domain was assessed as low, moderate, serious, critical, no information. Sensitivity analyses exclude serious, critical serious risk study (19).

### 2.6 Data analysis

For data analysis, we employed Stata 16.0 software (StataCorp LLC, Address:4905 Lakeway Dr, College Station, TX 77845). In our meta-analysis, we utilized the log OR (Odds Ratio), WMD (Weighted Mean Difference), and HR (hazard ratio) to synthesize the results across all included trials (20). A P-value threshold of less than 0.05 was set to determine statistical significance. Heterogeneity was evaluated using the Chi-square and q-test, with an I2 > 50% or a P-value < 0.10, suggesting notable diversity among studies. For these instances, we opted for a random-effects model.

## 3 Results

### 3.1 Description of study

**Figure 1:**
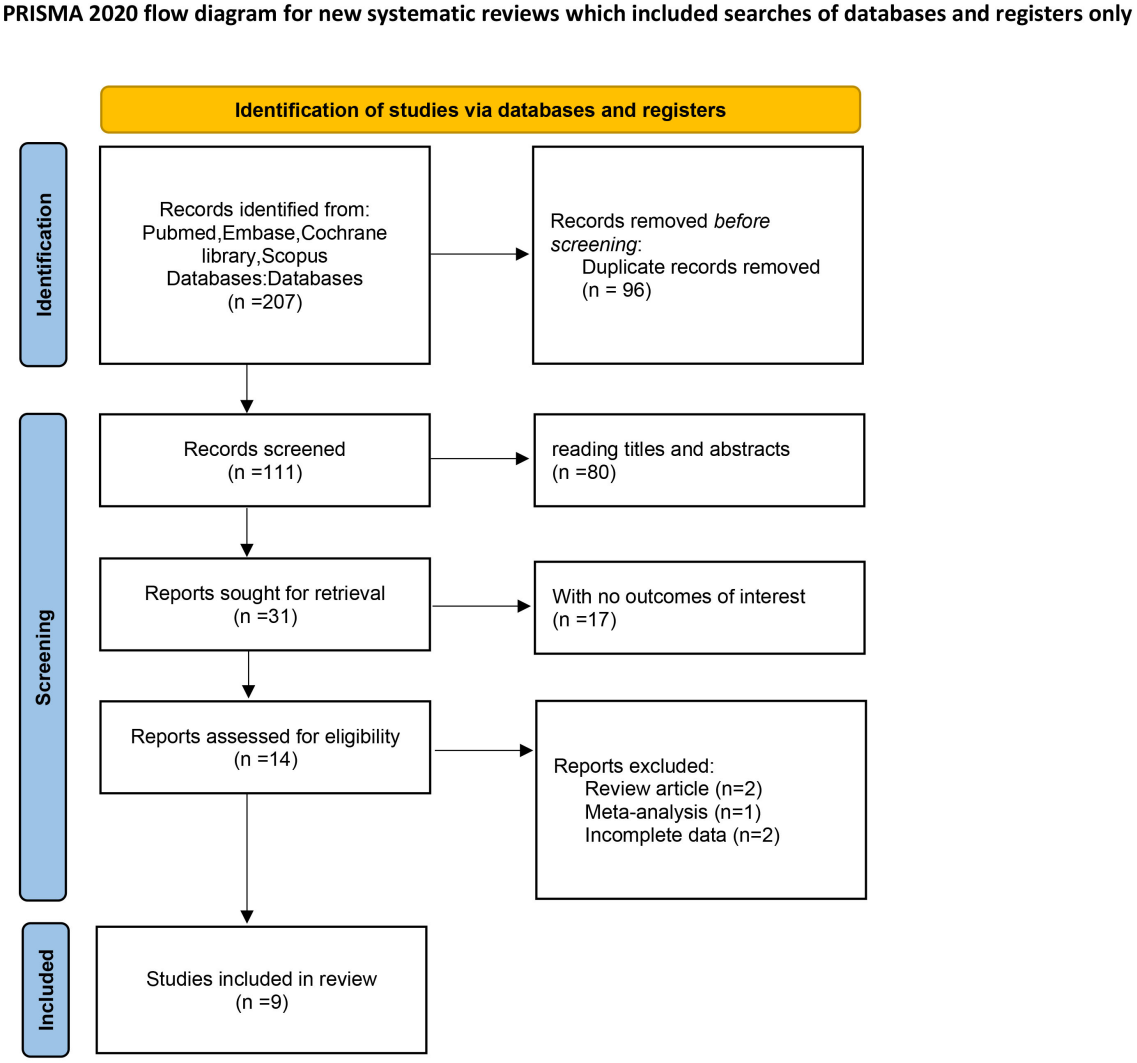
Flow diagram of the studies selection process. From: Page MJ, McKenzie JE, Bossuyt PM, Boutron I, Hoffmann TC, Mulrow CD, et al. The PRISMA 2020 statement: an updated guideline for reporting systematic reviews. *BMJ*. (2021) 372:n71. doi: 10.1136/bmj.n71

The authors searched 207 records from four databases. 96 duplicate studies were eliminated using document management software; 80 studies were excluded from reading titles and abstracts; 17 studies with no outcomes of interest, 2 systematic reviews, 1 Meta-analysis, and 2 incomplete data. A total of 9 studies involving 4295 patients were included in this meta-analysis (12–14, 21–26). In addition, the sample size was 37 ~ 1624. All 9 studies were retrospective studies. The screening process is shown in **Figure 1**, and the baseline characteristics of the included studies are shown in **Table 1**. 9 publications were published from 2020 – 2025.

**Table 1:** Baseline data for studies included in the meta-analysis.

Author	Year	Study type	Center	Sample (n)	Age	BMI^a^ (kg/m2)	Operative methods
Suoshi Jing (21)	2024	Retrospective	Single-Center	37	64.7	23.86	RARC^b^
Markus von Deimling (22)	2023	Retrospective	Single-Center	420	68	26	ORC^c^
Mahmoud Laymon (23)	2022	Retrospective	Single-Center	1624	57.9	27	ORC
Kai Li (24)	2022	Retrospective	Single-Center	340	66.4	NA	LRC^d^
Łukasz Zapała (25)	2022	Retrospective	Multi-center	304	68	25.8	ORC LRC
Pietro Piazza (13)	2022	Retrospective	Single-Center	366	71	26	RARC
P Baron (14)	2021	Retrospective	Multi-center	104	65.8	26.1	RARC
Jong Jin Oh (26)	2021	Retrospective	Multi-center	730	64.7	24.3	RARC
Giovanni E Cacciamani (12)	2020	Retrospective	Single-Center	370	70.3	27.6	RARC

BMI^a^, body mass index; RARC^b^, Robot-assisted laparoscopic radical cystectomy; ORC^c^, open radical cystectomy; LRC^d^, laparoscopic radical cystectomy.

### 3.2 Quality assessment

The quality of the cohort studies was evaluated using the modified Newcastle-Ottawa Scale, NOS score was 6 to 7 points. 9 studies were included in the assessment, all with a score of 6 or more in **Table 2**. The ROBINS-I tool was used to assess the risk of bias in the selected studies. 9 studies were included in the assessment, and the overall bias was “moderate risk” **Supplementary Table**.

**Table 2:** Quality score of included studies based on the NOS scale.

Study	Selection			Comparability			Exposure			Total stars
	^a^REC	^b^SNEC	^c^AE	^d^DO	^e^SC	^f^AF	^g^AO	^h^FU	^i^AFU	
Suoshi Jing	1	1	1		1	1	1	1		7
Markus von Deimling	1	1	1	1	1		1		1	7
Mahmoud Laymon	1	1	1	1	1			1	1	7
Kai Li	1	1	1	1			1	1	1	7
Łukasz Zapała	1	1	1	1		1	1	1		7
Pietro Piazza	1	1	1	1				1	1	6
P Baron	1	1	1	1	1	1	1			7
Jong Jin Oh	1	1	1	1				1	1	6
Giovanni E Cacciamani	1	1	1	1	1			1	1	7

^a^REC, representativeness of the cohort; ^b^SNEC, selection of the none posed cohort; ^c^AE, ascertainment of exposure; ^d^DO, demonstration that outcome of interest was not present at start of study; ^e^SC, study controls most important factors; ^f^AF, study controls for other important factors; ^g^AO, assessment of outcome; ^h^FU, follow-up long enough for outcomes to occur; ^i^AFU, adequacy of follow-up of cohort (≥ 80%).

### 3.3 Age

**Figure 2:**
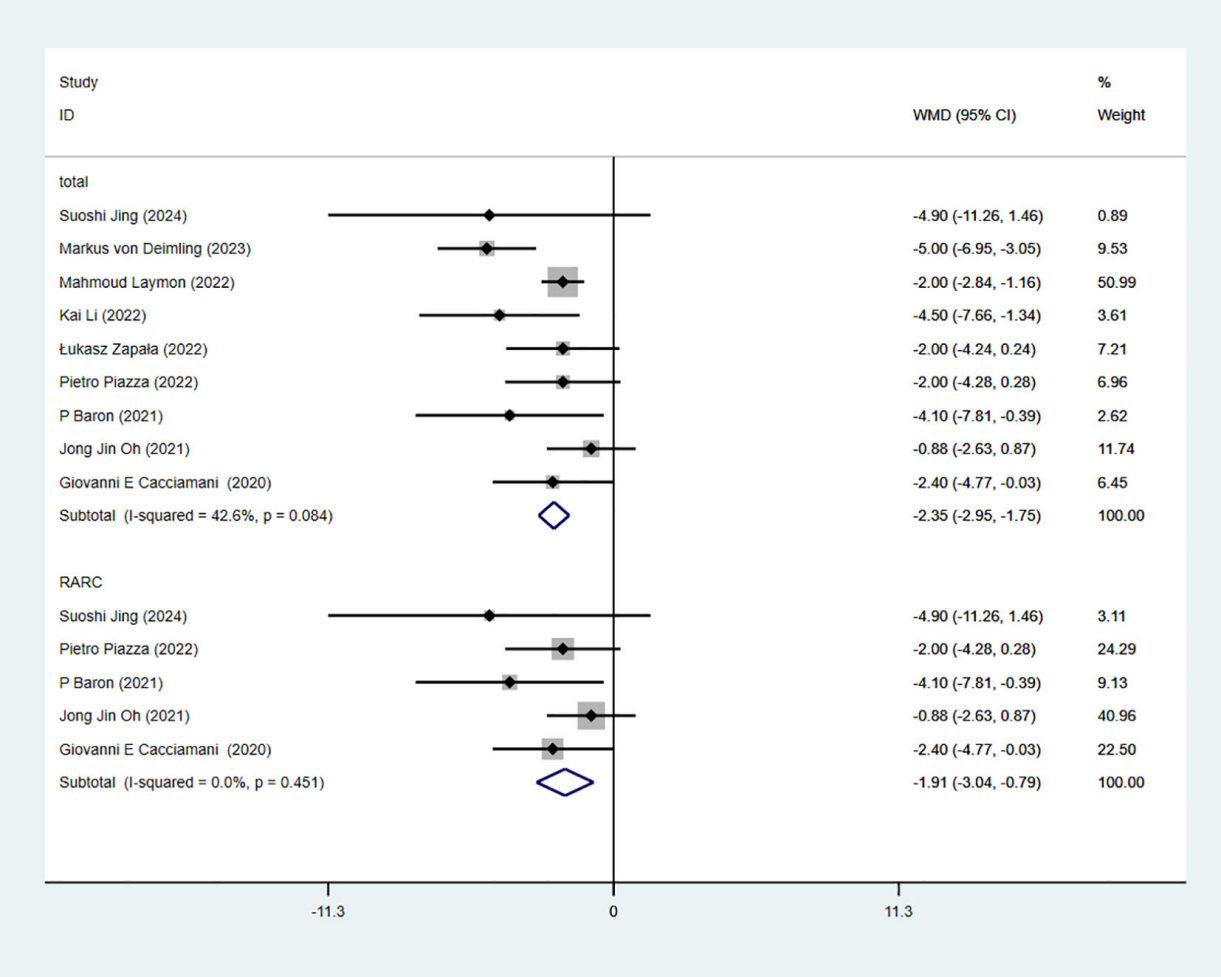
Forest plot and meta-analysis of age in RC-Pentafecta attained.

9 studies reported Age. The combined results demonstrated significant difference between the RC-Pentafecta attained group and RC-Pentafecta not attained group (WMD = -2.35, 95% CI [-2.95, -1.75], P < 0.05). A subgroup of robotic-assisted radical cystectomy (RARC) analysis showed that there were significant differences in between the RC-Pentafecta attained group and RC-Pentafecta not attained group (5 studies; WMD = -1.91, 95% CI [-3.04, -0.79], P < 0.05) (**Figure 2**).

### 3.4 Body mass index

**Figure 3:**
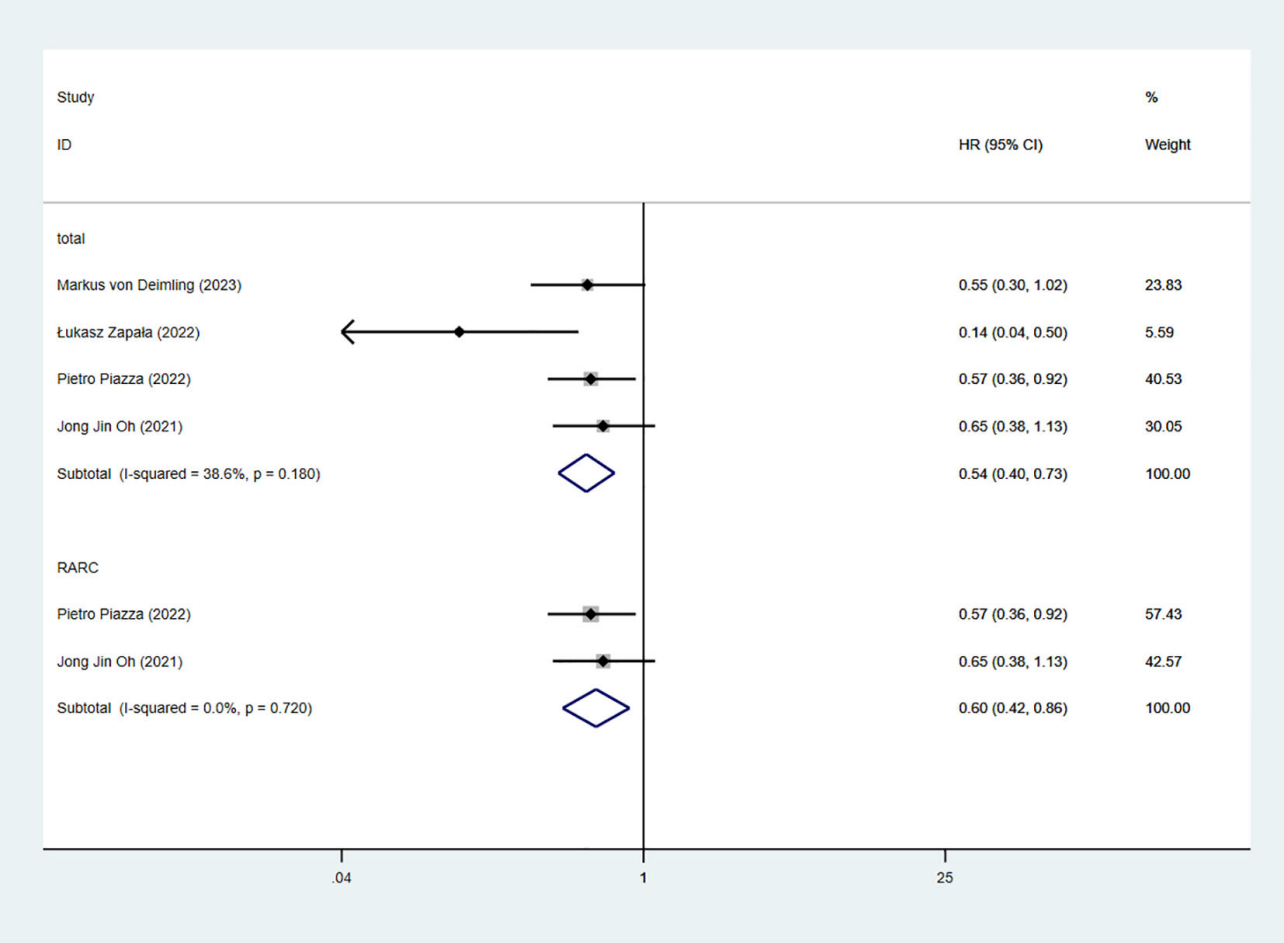
Forest plot and meta-analysis of BMI in RC-Pentafecta attained.

7 studies reported BMI. The combined results demonstrated no significant difference between the RC-Pentafecta attained group and RC-Pentafecta not attained group (WMD = -0.13, 95% CI [-0.94, 0.69], P > 0.05). A subgroup of RARC analysis showed that there were no significant differences between the RC-Pentafecta attained group and RC-Pentafecta not attained group (5 studies; WMD = -0.41, 95% CI [-1.46, 0.64], P > 0.05) (**Figure 3**).

### 3.5 Operative time

**Figure 4:**
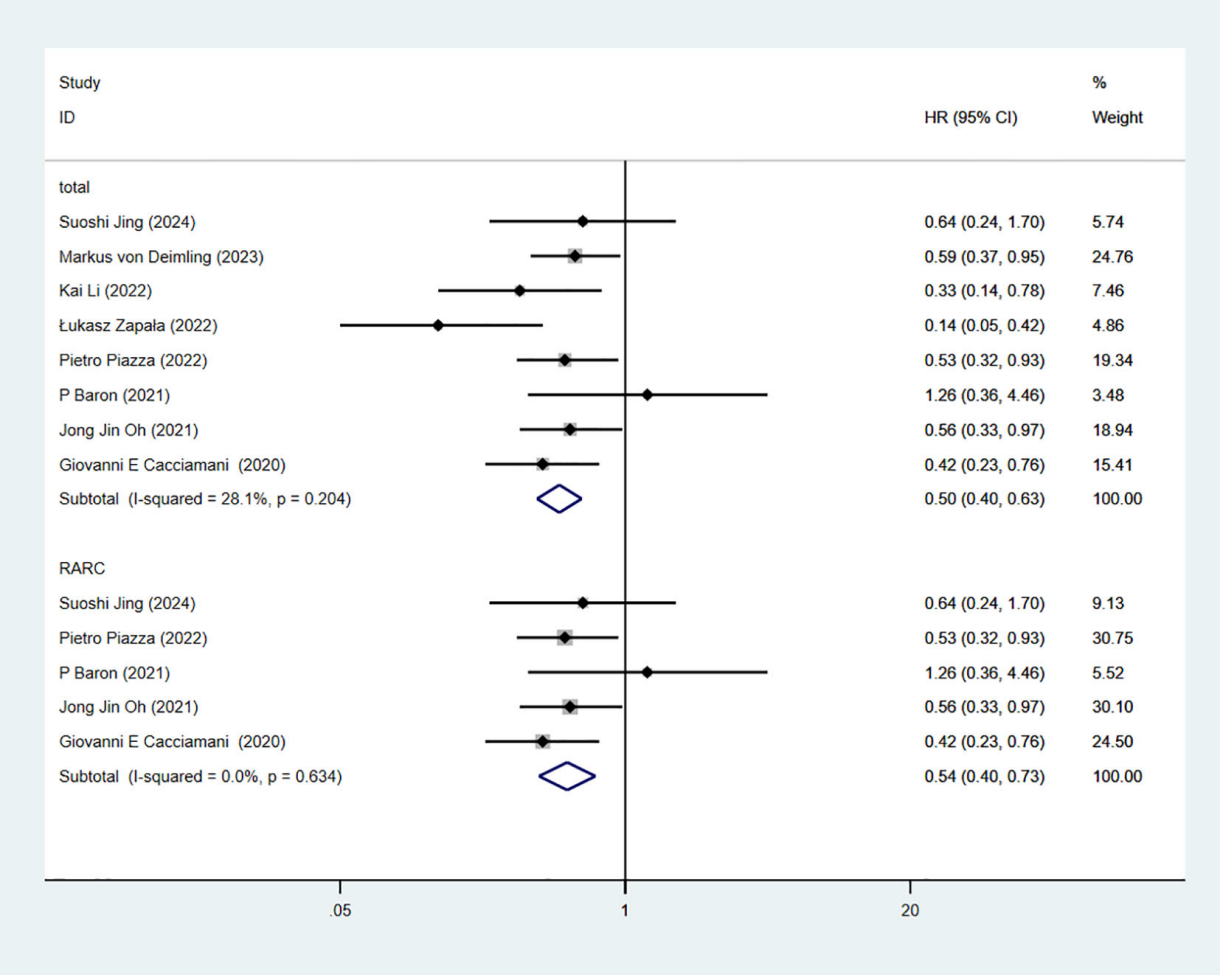
Forest plot and meta-analysis of OT in RC-Pentafecta attained.

7 studies reported OT. The combined results demonstrated no significant difference between the RC-Pentafecta attained group and RC-Pentafecta not attained group (WMD = -2.36, 95% CI [-21.15, 16.43], P > 0.05). A subgroup of RARC analysis showed that there were no significant differences in between the RC-Pentafecta attained group and RC-Pentafecta not attained group (5 studies; WMD = -4.68, 95% CI [-28.45, 19.09], P > 0.05) (**Figure 4**).

### 3.6 Length of hospital stay

**Figure 5:**
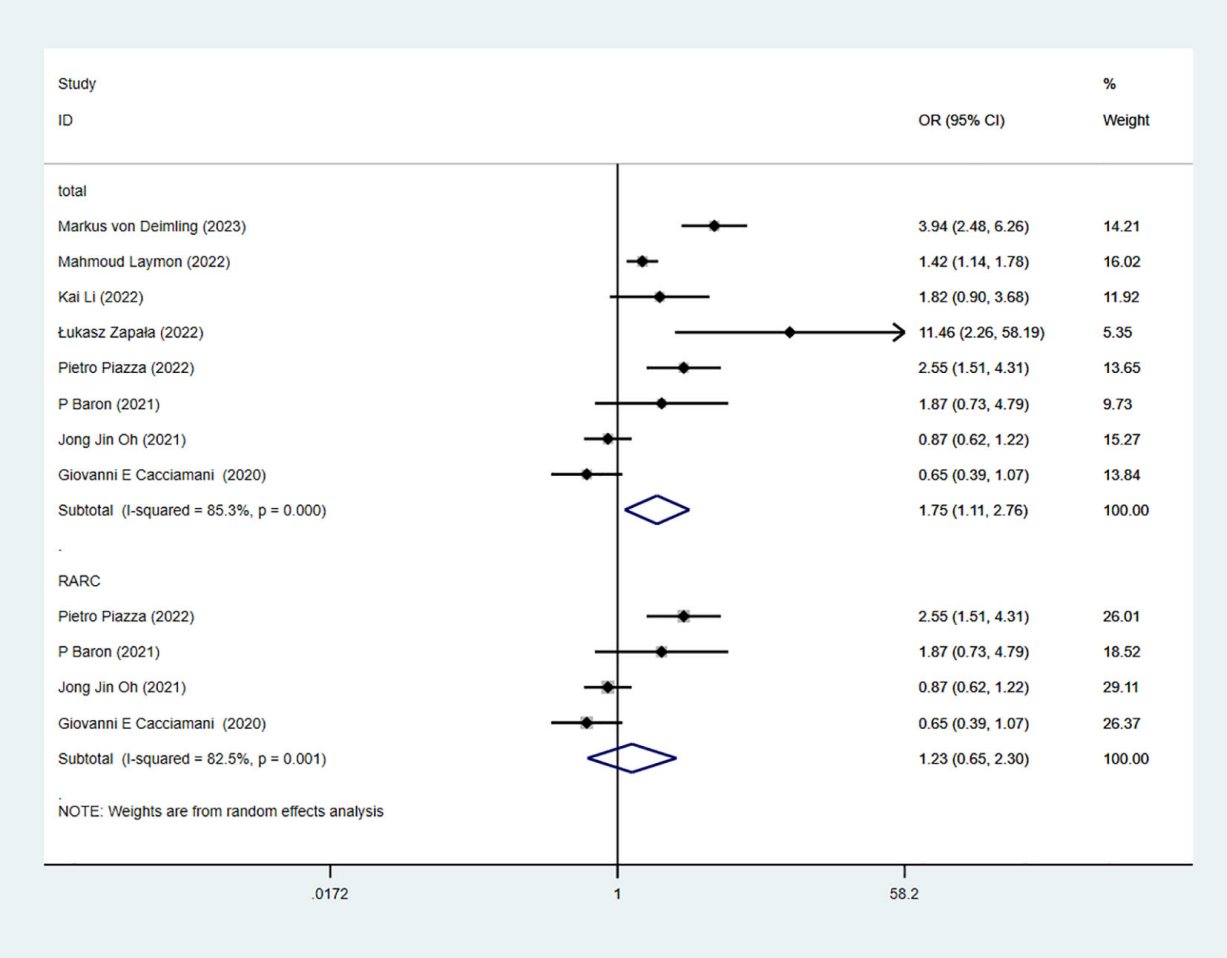
Forest plot and meta-analysis of LOS in RC-Pentafecta attained.

7 studies reported LOS. The combined results demonstrated significant difference between the RC-Pentafecta attained group and RC-Pentafecta not attained group (WMD = -1.34, 95% CI [-2.34, -0.33], P < 0.05). A subgroup of RARC analysis showed that there were significant differences in between the RC-Pentafecta attained group and RC-Pentafecta not attained group (5 studies; WMD = -1.63, 95% CI [-2.50, -0.77], P < 0.05) (**Figure 5**).

### 3.7 American Association of Anesthesiology score

**Figure 6:**
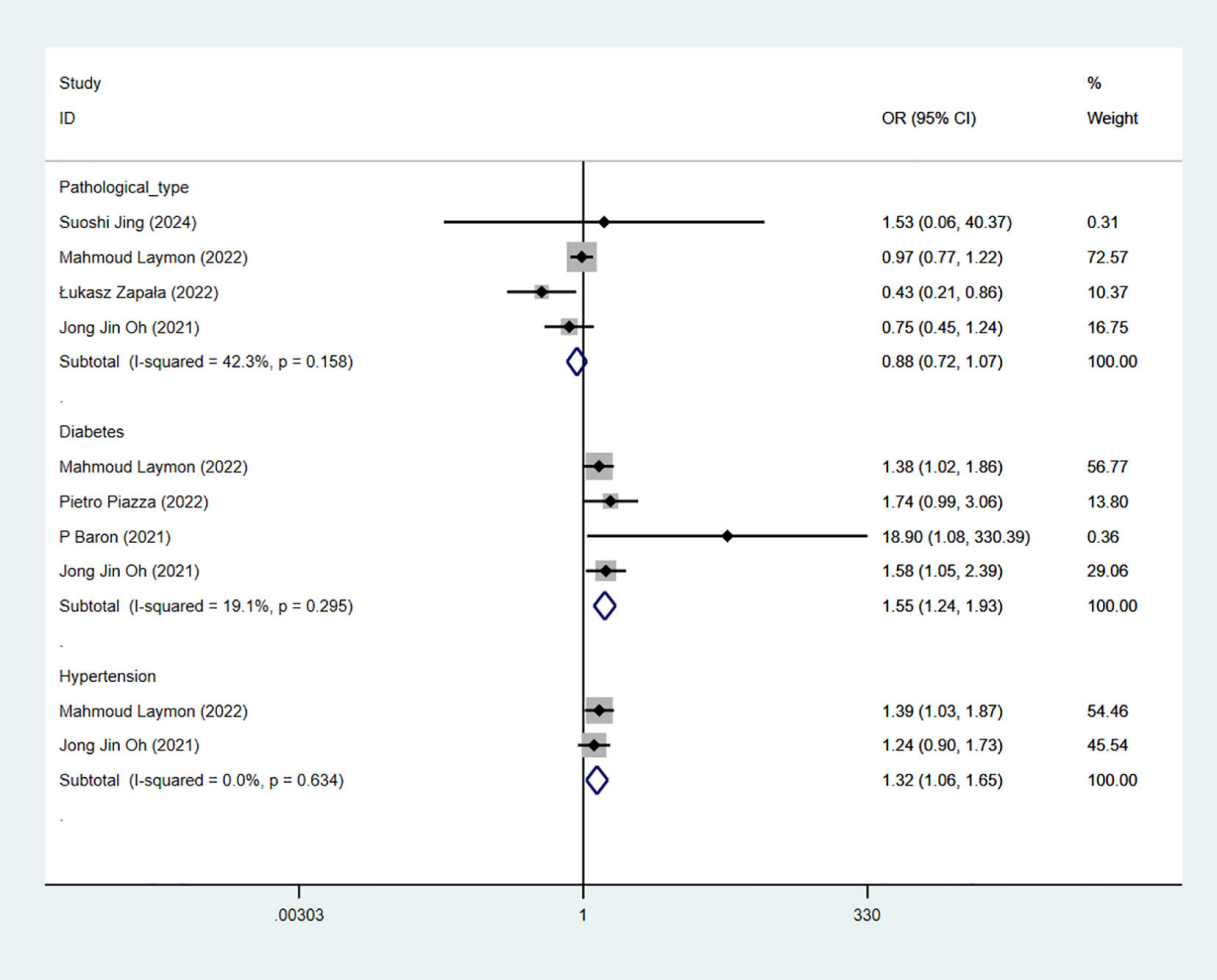
Forest plot and meta-analysis of ASA score, history of smoking, and NAC in RC-Pentafecta attained.

7 studies reported ASA score. The combined results demonstrated significant difference between the low-score-group and high-score-group in RC-Pentafecta (OR = 1.35, 95% CI [1.14, 1.59], P < 0.05) (**Figure 6**).

### 3.8 History of smoking

6 studies reported history of smoking. The combined results demonstrated no significant difference between the non-smoking-group and smoking-group in RC-Pentafecta (OR = 0.84, 95% CI [0.69, 1.03], P > 0.05) (**Figure 6**).

### 3.9 Neoadjuvant chemotherapy

7 studies reported NAC. The combined results demonstrated no significant difference between the NAC-group and non-NAC-group in RC-Pentafecta (OR = 0.89, 95% CI [0.70, 1.12], P > 0.05) (**Figure 6**).

### 3.10 Pathological type

**Figure 7:**
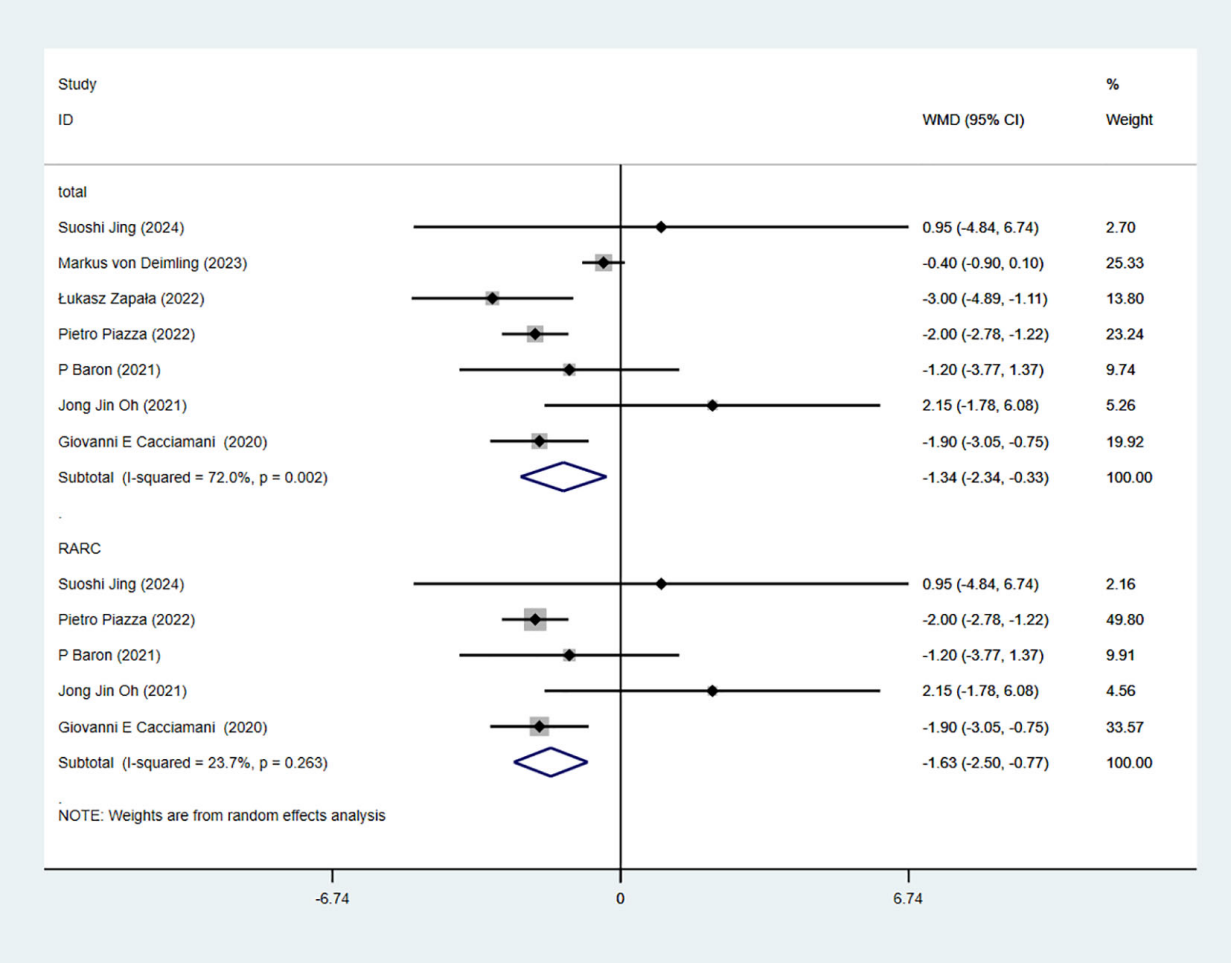
Forest plot and meta-analysis of pathological type, diabetes, and hypertension in RC-Pentafecta attained.

4 studies reported pathological type. The combined results demonstrated no significant difference between the urothelium carcinoma (UC) group and non-UC group in RC-Pentafecta (OR = 0.88, 95% CI [0.72, 1.07], P > 0.05) (**Figure 7**).

### 3.11 Diabetes

4 studies reported diabetes. The combined results demonstrated significant difference between the non-diabetes-group and diabetes-group in RC-Pentafecta (OR = 1.55, 95% CI [1.24, 1.93], P < 0.05) (**Figure 7**).

### 3.12 Hypertension

2 studies reported hypertension. The combined results demonstrated significant difference between the non-hypertension-group and hypertension-group in RC-Pentafecta (OR = 1.32, 95% CI [1.06, 1.65], P < 0.05) (**Figure 7**).

### 3.13 Pathological T-stage

**Figure 8:**
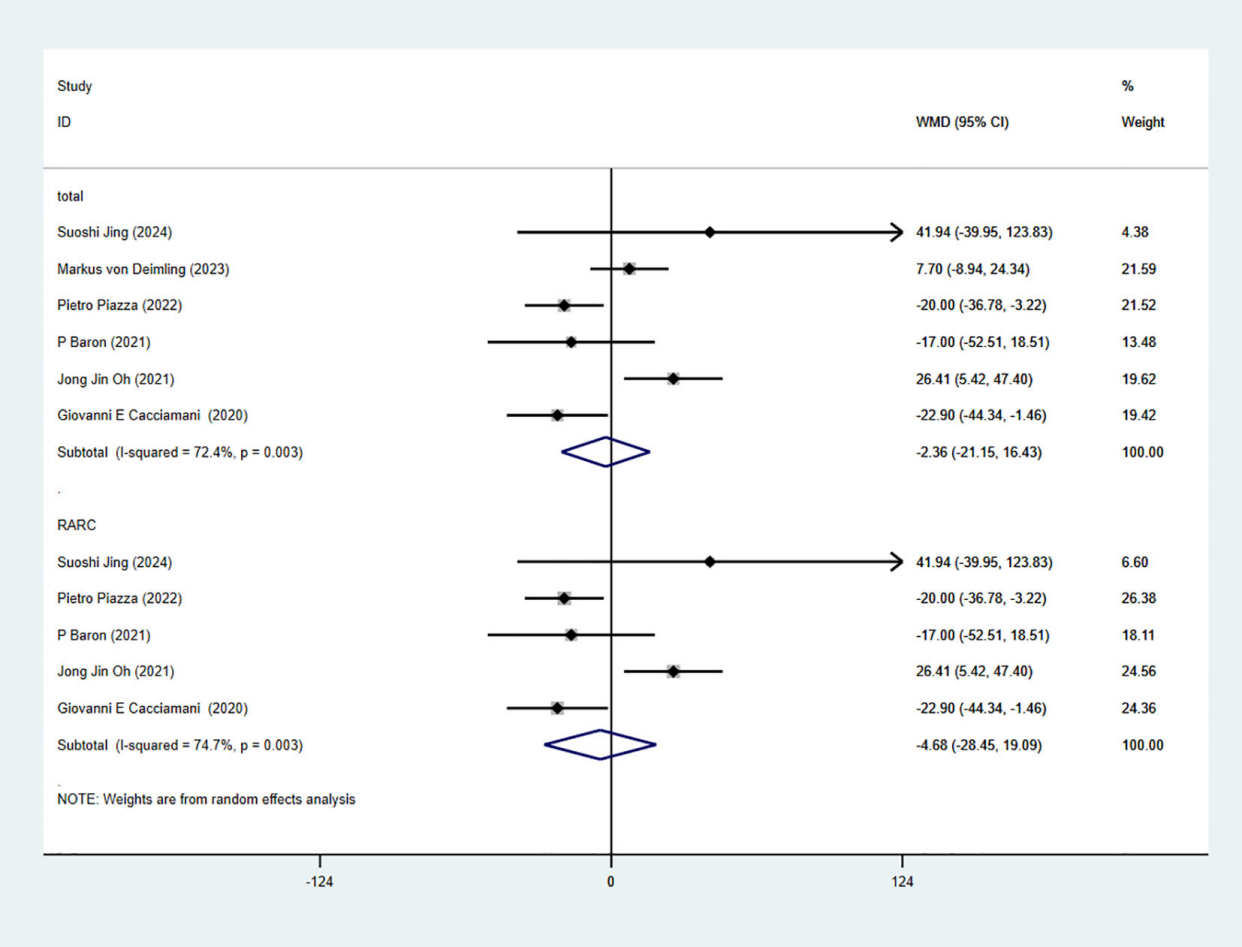
Forest plot and meta-analysis of pT-stage (when pT≥1, pT≥2) in RC-Pentafecta attained.

**Figure 9:**
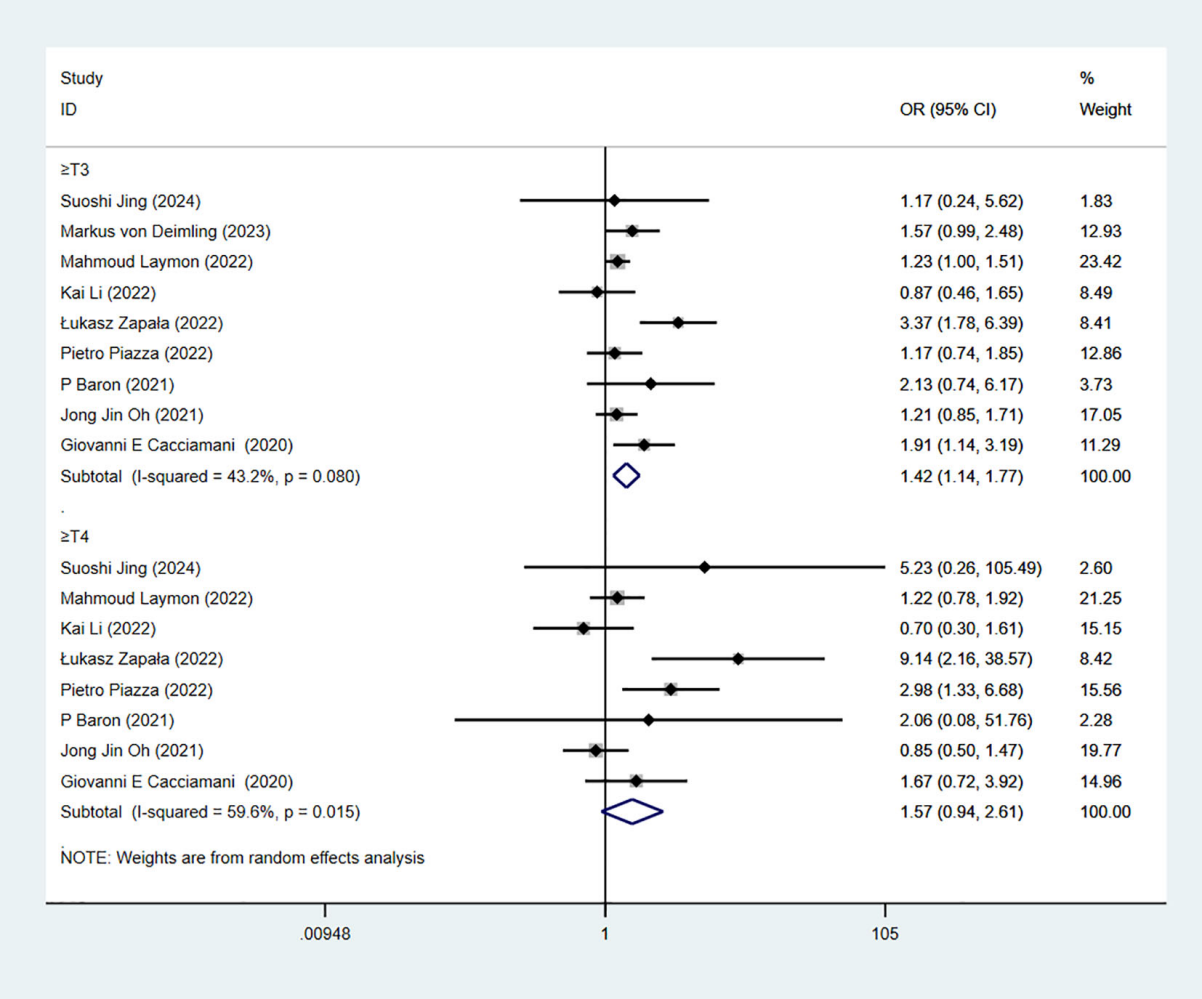
Forest plot and meta-analysis of pT-stage (when pT≥3, pT≥4) in RC-Pentafecta attained.

pT-stage is based on postoperative tumor histopathological examination to assess the depth of bladder cancer invasion into the bladder wall and surrounding tissues, reflecting local tumor progression. 9 studies reported pT-stage. The combined results demonstrated significant difference when pT≥1 in RC-Pentafecta (7 studies; OR = 1.44, 95% CI [1.12, 1.85], P < 0.05). The combined results demonstrated significant difference when pT≥2 in RC-Pentafecta (9 studies; OR = 1.25, 95% CI [1.07, 1.45], P < 0.05). (**Figure 8**). The combined results demonstrated significant difference when pT≥3 in RC-Pentafecta (9 studies; OR = 1.42, 95% CI [1.14, 1.77], P < 0.05). The combined results demonstrated no significant difference when pT≥4 in RC-Pentafecta (8 studies; OR = 1.57, 95% CI [0.94, 2.61], P > 0.05) (**Figure 9**).

### 3.14 Pathological N-stage

**Figure 10:**
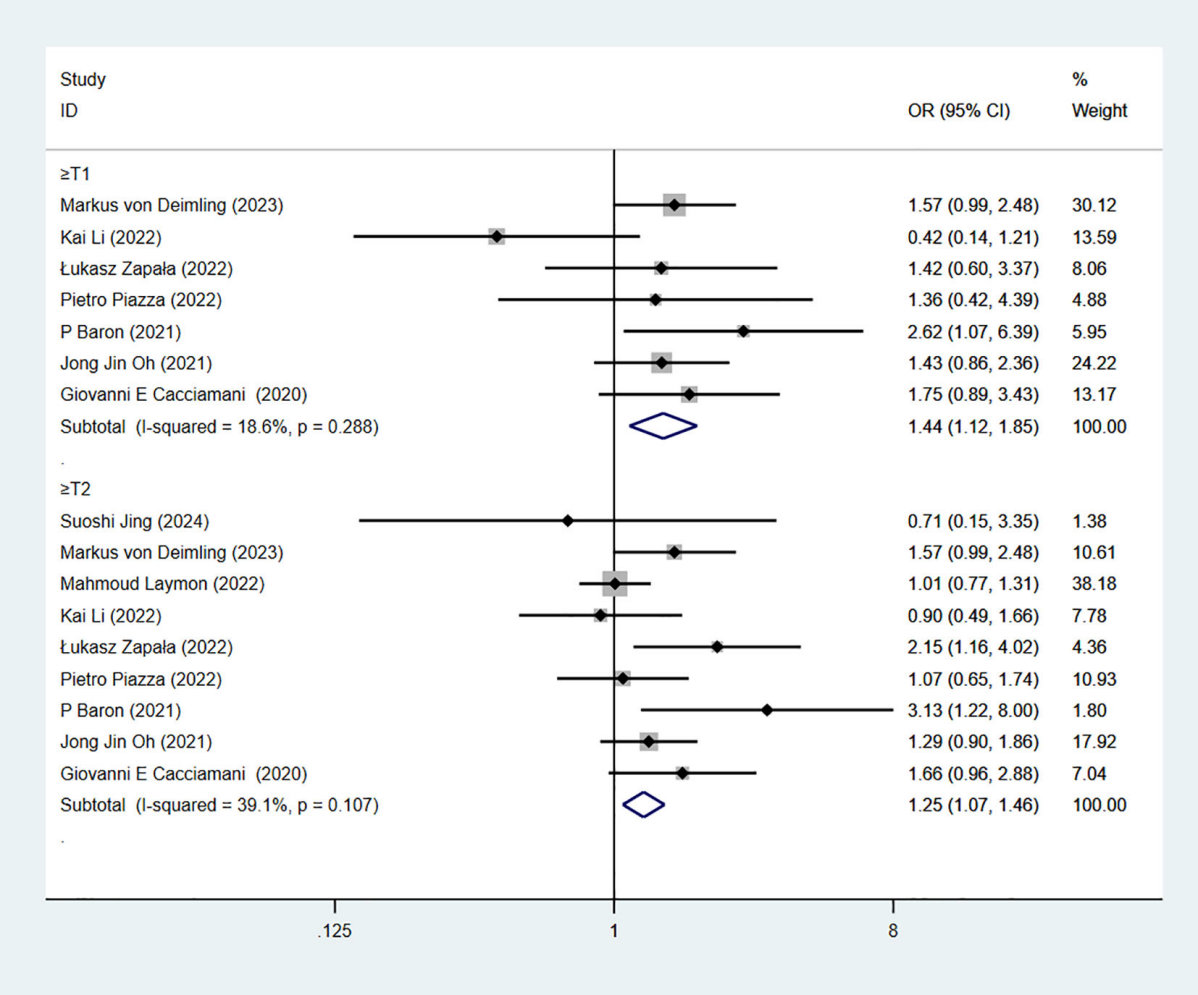
Forest plot and meta-analysis of pN-stage in RC-Pentafecta attained.

pN-stage, based on postoperative lymph node pathological examination, assesses whether regional or distant lymph node metastasis has occurred in bladder cancer, reflects the extent of tumor spread, and is a key indicator for prognosis judgment. 9 studies reported pN-stage. The combined results demonstrated significant difference when pN≥1 in RC-Pentafecta (9 studies; OR = 1.35, 95% CI [1.15, 1.59], P < 0.05). The combined results demonstrated significant difference when pN≥2 in RC-Pentafecta (8 studies; OR = 1.32, 95% CI [1.06, 1.64], P < 0.05). The combined results demonstrated no significant difference when pN≥3 in RC-Pentafecta (4 studies; OR = 1.17, 95% CI [0.52, 2.64], P > 0.05) (**Figure 10**).

### 3.15 Type of urinary diversion

**Figure 11:**
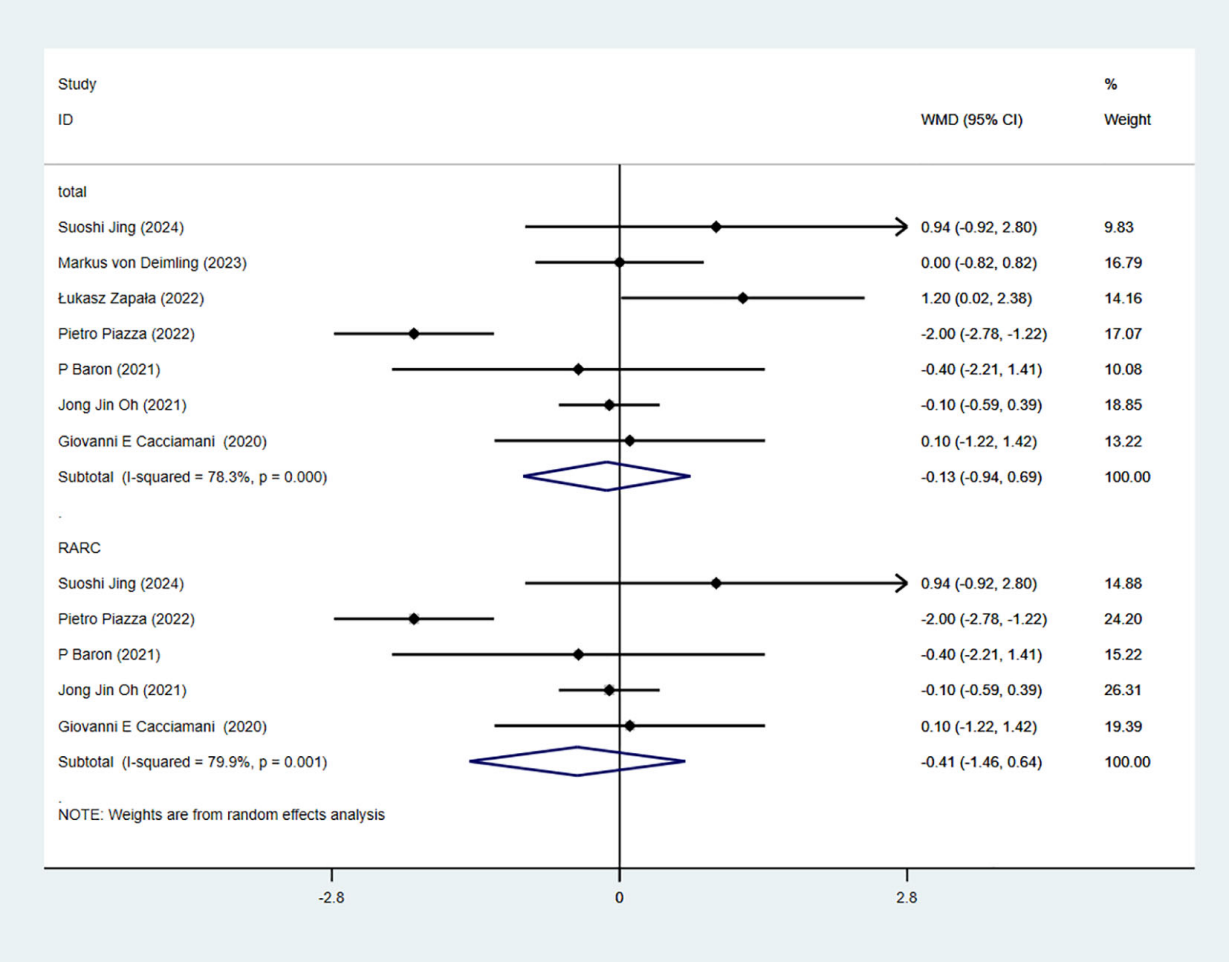
Forest plot and meta-analysis of type of UD in RC-Pentafecta attained.

8 studies reported type of UD. The combined results demonstrated significant difference between the continent-UD-group and incontinent-UD-group in RC-Pentafecta (OR = 1.75, 95% CI [1.11, 2.76], P < 0.05). A subgroup of RARC analysis showed that there were significant differences between the continent-UD-group and incontinent-UD-group group (4 studies; OR = 1.23, 95% CI [0.65, 2.30], P > 0.05) (**Figure 11**).

### 3.16 Overall survival

**Figure 12:**
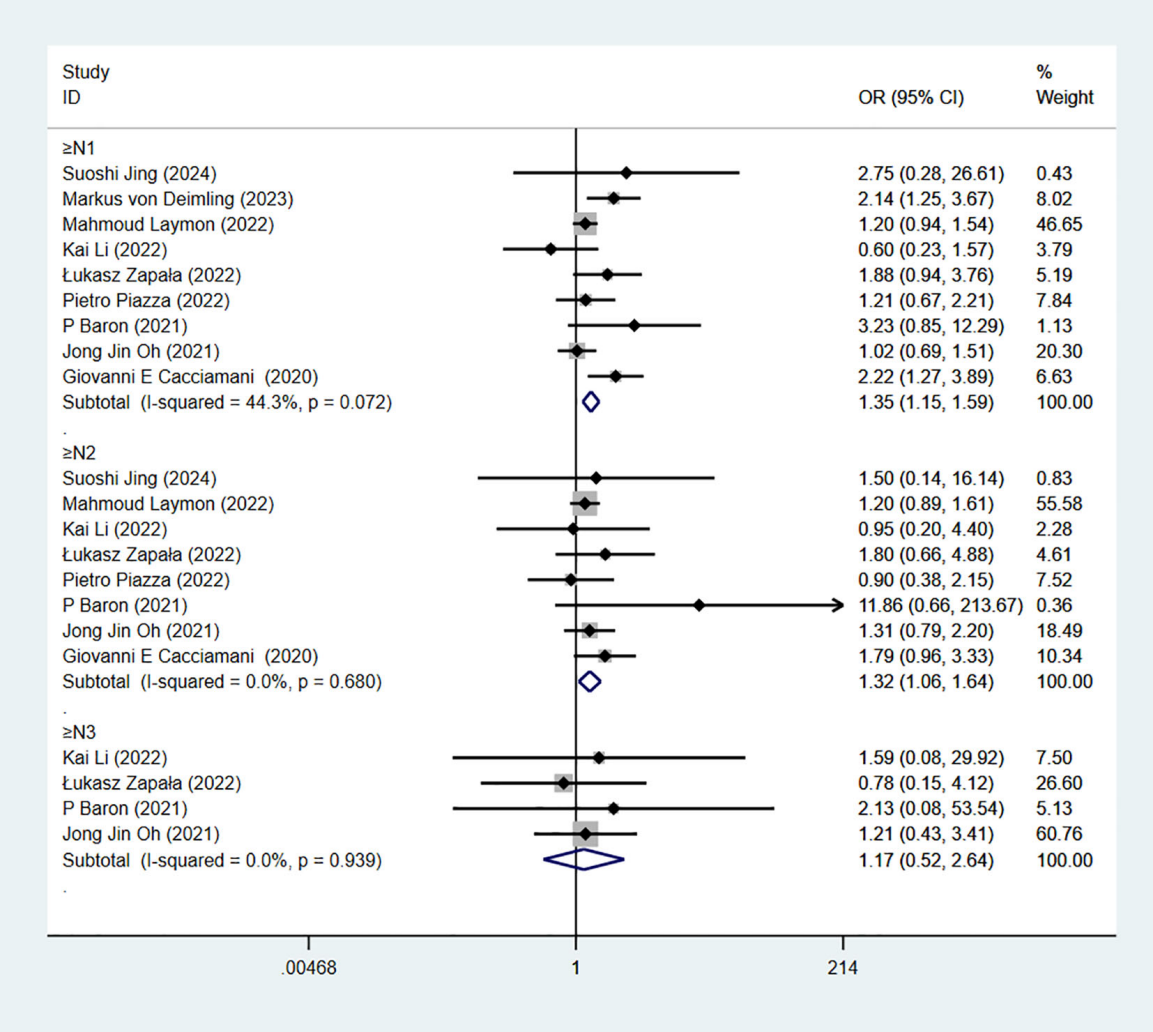
Forest plot and meta-analysis of type of OS in RC-Pentafecta attained.

8 studies reported OS. The combined results demonstrated significant difference between the RC-Pentafecta attained group and RC-Pentafecta not attained group (HR = 0.50, 95% CI [0.40, 0.63], P < 0.05). A subgroup of RARC analysis showed that there were significant differences in between the RC-Pentafecta attained group and RC-Pentafecta not attained group (5 studies; HR = 0.54, 95% CI [0.40, 0.73], P < 0.05) (**Figure 12**).

### 3.17 Cancer-specific survival

**Figure 13:**
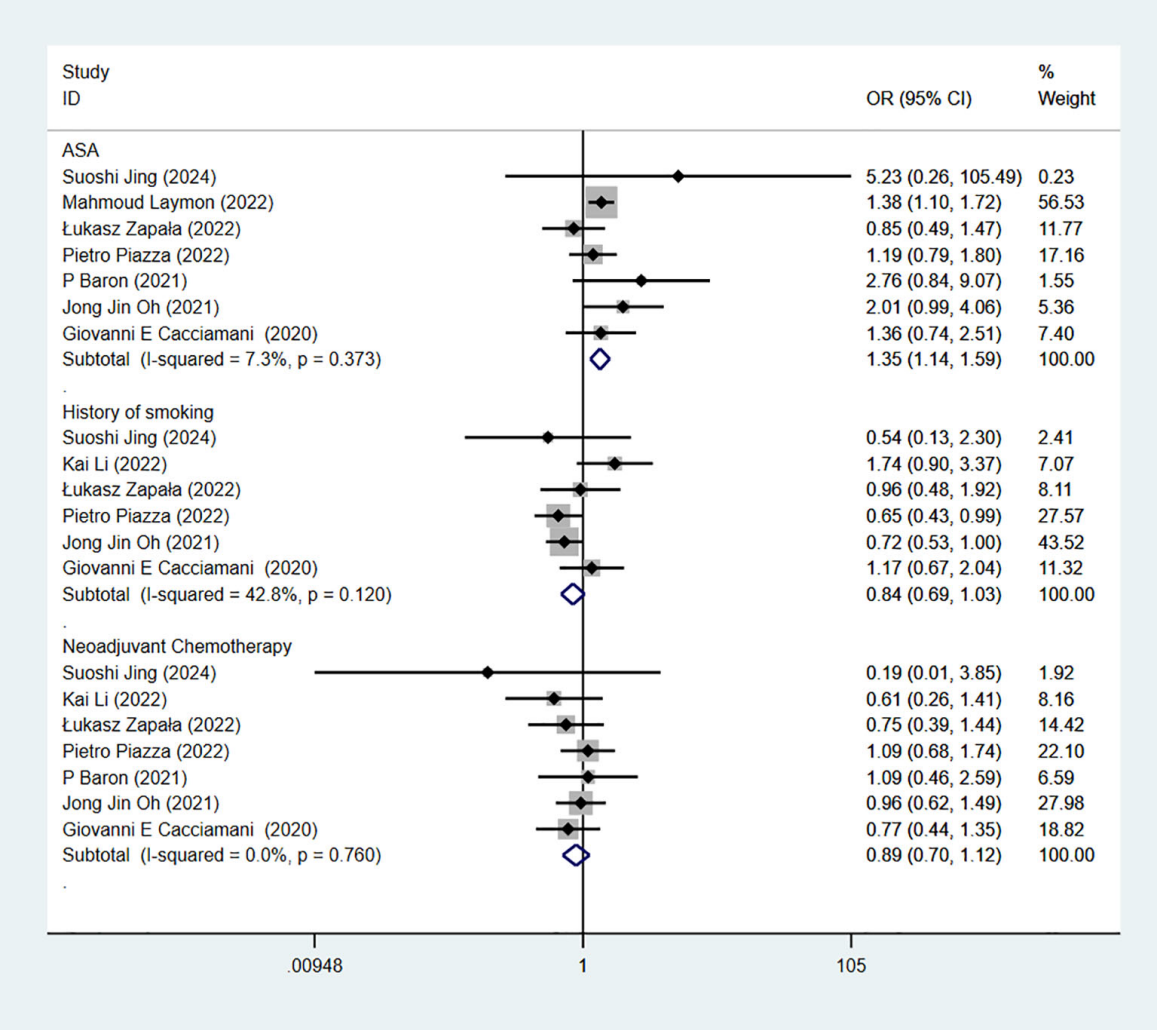
Forest plot and meta-analysis of type of CSS in RC-Pentafecta attained.

4 studies reported CSS. The combined results demonstrated significant difference between the RC-Pentafecta attained group and RC-Pentafecta not attained group (HR = 0.54, 95% CI [0.40, 0.73], P < 0.05). A subgroup of RARC analysis showed that there were significant differences in between the RC-Pentafecta attained group and RC-Pentafecta not attained group (2 studies; HR = 0.60, 95% CI [0.42, 0.86], P < 0.05) (**Figure 13**).

## 4 Sensitivity analysis

We used sensitivity analyses to track sources of heterogeneity for each outcome measure. The results showed that the pathological type was a stable source of heterogeneity.

## 5 Discussion

Through a meta-analysis of 9 studies, patients who achieved the RC-Pentafecta criteria had a significant advantage in OS and CSS. In addition, Age, LOS, ASA score, combined diabetes, combined hypertension, UD type, pT and pN, and RC-Pentafecta showed significant differences between the two groups. BMI, OT, pathological type, smoking history, and combined neoadjuvant chemotherapy perioperative indicators showed no significant difference between the two groups.

Age showed significant differences in this study. Generally, young patients have no serious underlying diseases, more robust immune function, stronger postoperative recovery ability, and lower incidence of postoperative complications (27, 28). This makes it easier for them to meet the RC-Pentafecta criteria. Although BMI is a potential influencing factor for surgical risk, it did not show a significant difference in this study. The reason may be the complex effect of BMI on postoperative recovery. BMI that is too high or too low may increase the difficulty of surgery and postoperative complications (29, 30). There was no significant difference in OT between the two groups. Although longer surgical duration may increase the risk of postoperative complications, LN resection to achieve RC-Pentafecta criteria may require longer OT. Therefore, the OT did not show a significant difference between the two groups (31). LOS is an important indicator of postoperative recovery status, and the RC-Pentafecta attained group had significantly shorter LOS. LOS is closely related to the incidence of complications and the overall health status of patients. Shorter LOS means fewer complications and good recovery (32). ASA scores also showed significant differences between the RC-Pentafecta attained and non-achieved groups. The higher the ASA score, the worse the patient’s general health status and the greater the risk of surgery. Patients with low ASA scores usually have no serious underlying diseases, better surgical tolerance, and faster postoperative recovery, so it is easier to achieve RC-Pentafecta criteria (33, 34). Smoking history and RC-Pentafecta did not show significant differences. Although smoking is a known risk factor for BCa (35), its effect on complications may be limited in the short-term recovery from surgery. One study suggested that exposure to smoking and duration of smoking cessation were associated with postoperative recurrence and progression of RC (36). Unfortunately, the included studies did not mention the duration of smoking cessation or exposure level. Therefore, the relationship between smoking history and RC-Pentafecta may be confirmed by more studies in the future. NAC did not show a significant difference in the RC-Pentafecta. Although NAC can reduce tumor burden and improve survival in some patients, in the short-term perioperative period, it may increase the risk of postoperative complications, such as myelosuppression or infection, which may offset its advantage in tumor control (37, 38). In addition, BCa with a higher clinical stage is more likely to receive NAC (39), which means NAC patients may have worse perioperative and oncologic outcomes. There was no significant difference in pathological type between the two groups. This may be because the majority of patients had UC (21, 25, 26), which is the most common type of BCa (35). The sample sizes of other rare pathological types were small and did not yield sufficient statistical differences. Diabetes mellitus and hypertension were common underlying diseases. Patients with diabetes and hypertension face a higher risk of complications during postoperative recovery, such as infection and cardiovascular complications, which increase the incidence of perioperative mortality and complications (40–42). pT and pN stages indicate tumor aggressiveness and disease severity. These patients have a high operative difficulty and a high probability of postoperative recurrence, which affects the OS, RFS, and CSS. No statistical difference was shown when pT≥4 and pN≥3, but there were fewer patients with pT≥4 and pN≥3, and this result may need to be confirmed with more samples. The Type of UD had a significant difference in the RC-Pentafecta. Incontinent-UD brought more incontinence-related complications, such as skin irritation and infection due to urine leakage (43). The OS and CSS of the RC-Pentafecta attained group was significantly better than that of the non-attained group. STSMs are a very important RC-Pentafecta criteria. Patients who achieved STSMs meant that the tumor had been completely removed during operation, leaving no residual tumor tissue. STSMs was significantly associated with lower RFS (44). Adequate LN dissection can help to determine the tumor stage and guide the subsequent treatment accurately. It can also remove potential metastatic LN, thereby reducing the risk of distant metastasis (45). The RC-Pentafecta group had no major complications, a smoother postoperative recovery, and better overall health status. The NOS scores of all studies were of medium to high quality, but the overall bias of ROBINS-I was “moderate risk”. The core reason for the difference lies in the different assessment objectives and dimension designs of the two tools. A high NOS score only indicates that the study complies with the design norms of cohort studies. ROBINS-I more accurately identified the biases in retrospective studies, thus resulting in the outcome that “NOS is of high quality but ROBINS-I is moderately biased”. There is no contradiction between the two, but rather complementary evaluations of research quality from different dimensions.

Although this study discusses perioperative predictors of achieving RC-Pentafecta and the potential of RC-Pentafecta to predict oncological outcomes, there are certain limitations. (1) Only retrospective studies were included in this study. Although the quality of the studies was rigorously assessed, there may still be selection bias and the inherent limitations of retrospective studies. (2) Some studies do not fully consider the quality of life and functional recovery of patients after surgery, which also have an important impact on the long-term survival of patients. (3) Although the effect of smoking history on the results was discussed, the exposure to smoking and the duration of smoking cessation was not further discussed. (4) Due to the fact that the original studies included did not systematically record and report some perioperative indicators during the data collection stage, this study is temporarily unable to incorporate them into the analysis of Pentafecta influencing factors, such as preoperative blood loss and blood transfusion requirements, etc. More prospective and multi-center studies should be included in future studies to verify further the perioperative predictions of RC-Pentafecta and the ability of RC-Pentafecta to predict oncological outcomes.

## 6 Conclusion

RC-Pentafecta is a valuable criterion that can effectively predict OS and CSS in patients after RC. Age, perioperative health status, and pathological stage are important predictors of RC-Pentafecta.

## Data Availability

The original contributions presented in the study are included in the article/**Supplementary Material**. Further inquiries can be directed to the corresponding author.
