# Supplementary figures and images for: Predictors and oncological outcomes of achieving Pentafecta in radical cystectomy: a meta-analysis
Source: Front Oncol. 2026 Jan 8;15:1682830. doi: 10.3389/fonc.2025.1682830 (PMC12823531; doi:10.3389/fonc.2025.1682830)

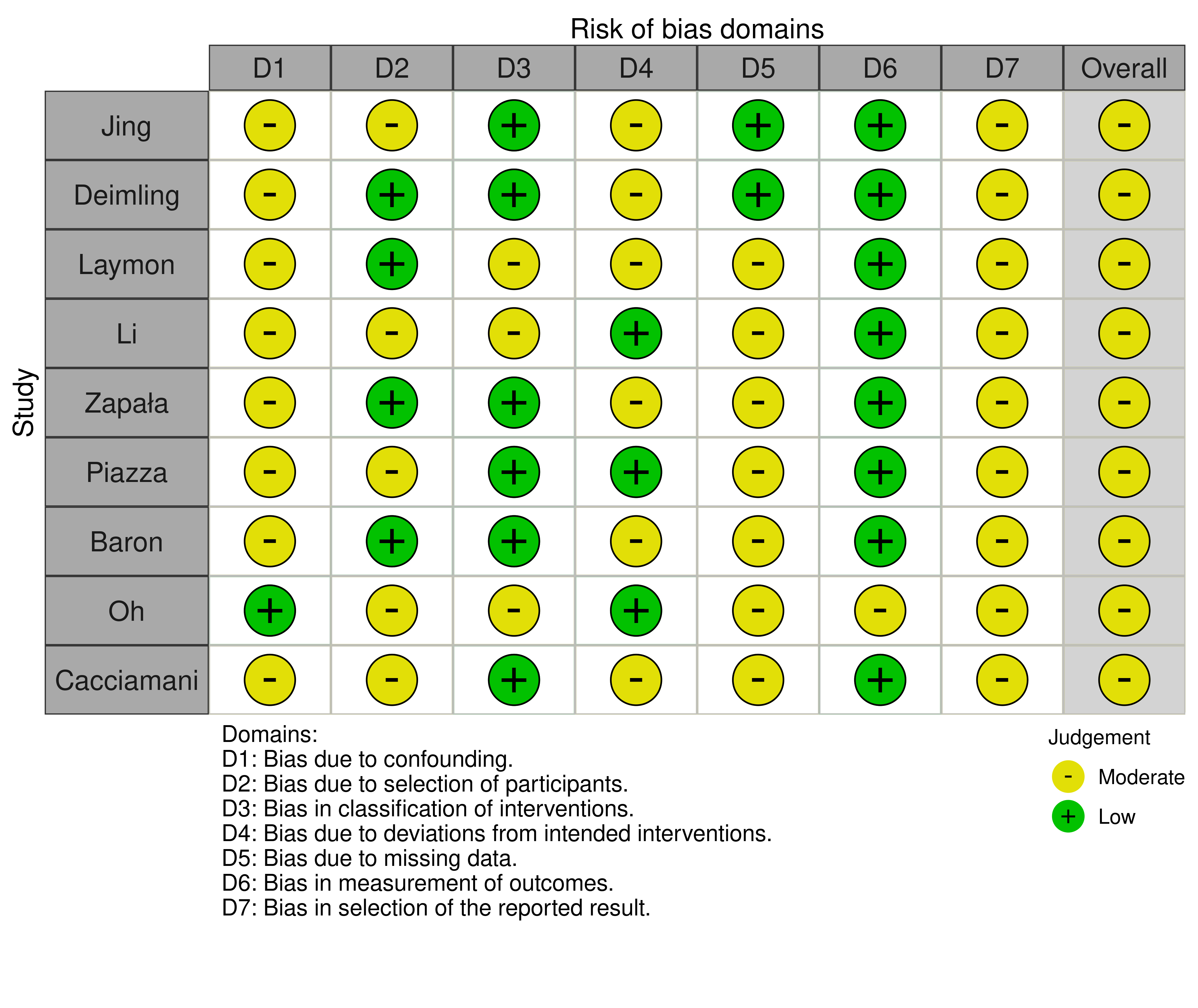

Supplement: Supplementary file 1 [file Image1.png]
